# Hypergraph and network flow-based quality function deployment

**DOI:** 10.1016/j.heliyon.2022.e12263

**Published:** 2022-12-09

**Authors:** János Abonyi, Tímea Czvetkó

**Affiliations:** ELKH-PE Complex Systems Monitoring Research Group, University of Pannonia, Egyetem str. 10, H-8200 Veszprém, Hungary

**Keywords:** Quality function deployment, Product development, Hypergraph, Minimum cost flow, Linear programming

## Abstract

Quality function deployment (QFD) has been a widely-acknowledged tool for translating customer requirements into quality product characteristics based on which product development strategies and focus areas are identified. However, the QFD method considers the correlation and effect between development parameters, but it is not directly implemented in the importance ranking of development actions. Therefore, the cross-relationships between development parameters and their impact on customer requirement satisfaction are often neglected. The primary objective of this study is to make decision-making more reliable by improving QFD with methods that optimize the selection of development parameters even under capacity or cost constraints and directly implement cross-relationships between development parameters and support the identification of interactions visually. Therefore, QFD is accessed from two approaches that proved efficient in operations research. 1) QFD is formulated as a network flow problem with two objectives: maximizing the benefits of satisfying customer needs using linear optimization or minimizing the total cost of actions while still meeting customer requirements using assignment of minimum cost flow approach. 2) QFD is represented as a hypergraph, which allows efficient representation of the interactions of the relationship and correlation matrix and the determination of essential factors based on centrality metrics. The applicability of the methods is demonstrated through an application study in developing a sustainable design of customer electronic products and highlights the improvements' contribution to different development strategies, such as linear optimization performed the best in maximizing customer requirements' satisfaction, assignment as minimum cost flow approach minimized the total cost, while the hypergraph-based representation identified the indirect interactions of development parameters and customer requirements.

## 1 Introduction

There is great competition between companies on the market to best convert customers' requirements and expectations into a quality product or service so that resources are utilized the most effectively. Thereby, product planning and design are critical in terms of gaining competitive advantage [1] and quality strategy should be the priority for companies [2]. The continuously changing customer requirements demand rapid re- or even pro-action to their needs, quality and systems thinking [3] which facilitates flexibility in companies' operation and the ability for quick adaptation [1]. To expect changes in the customer requirements, satisfy their demand while meeting the development goals, and manage (optimal) resource allocation, integrated methods are highly needed for better accuracy [4].

Quality Function Deployment (QFD) is a widely acknowledged standardized method [5], a “concept that provides a means of translating customer requirements into the appropriate technical requirements for each stage of product development and production (i.e., marketing strategies, planning, product design and engineering, prototype evaluation, production process development, production, sales)” [6]. This method reveals the relationship between customer requirements and the development parameters of the products in a relationship matrix, which defines the contribution of development parameters to satisfy customer needs. The method provides additional information for the development team about the interaction between the development parameters, representing how a change in the development parameter would affect the others. However, this correlation between the development parameters is considered but not implemented directly in calculating importance ranking. To analyze this manually can be time-consuming and complicated since the structure of QFD can be quite extensive with many requirements and development parameters, even in the case of a simple product [7].

Therefore, this study provides methods that support the engineers work, by implementing the correlation matrix into the calculation, and analyze cross-relationships to better identify focus and investment areas to best meet the requirements in a way that resources are utilized effectively. In this regard, QFD can be considered as a cost-benefit analysis [8], where we want to know what would be the benefit of our investments - how much customer requirements are satisfied based on the selected development parameters. The investments or resource allocations are based on the importance ranking of development parameters, which specify what would be its benefit, for example, minimum cost or highest efficiency of actions to meet requirements.

The primary objective of this study is to support product planning and design as well as resource allocation strategy making by introducing methods to improve QFD with the following two fundamental aspects:

• The direct implementation of the correlation matrix of development parameters into the calculation and evaluation of QFD. In this way, better identification of cross-correlations and allocation of resources can be achieved, thereby better product planning, which fulfills the customer requirements under possible cost and capacity constraints.
• The ability to define either the importance of customer requirements or the importance of development parameters is needed. It can provide additional information about the element's importance and objectively serves as cross-validation.

We propose methods that satisfy the above aspects and access the whole House of Quality (HOQ) including the correlation matrix of development parameters, the relationship matrix, the weights of requirements, and the actions over the development parameters (importance). These methods considering QFD as a 1) network flow optimization problem and a 2) hypergraph-based analysis.

1) Network flow-based optimization problem
  The network flow-based analysis is often used in operations research and has proved to be efficient in supporting optimal resource allocation in QFD [9]- which development parameters are worth investing in to meet customer requirements under given constraints. The following development strategies can be followed:
  **–** Maximize total efficiency: QFD is formulated as a linear programming problem with a reverse approach to the HOQ. Linear programming can be a potential solution to a network flow problem. We propose optimizing the selection of development parameters that we would invest in so customer requirements are most satisfied. In case of limited resources, the fulfillment of customer requirements should be maximized, while the cost of actions should not exceed the capacity.
  **–** Minimize total cost of actions: QFD is considered an assignment as a minimum cost flow problem. The resource allocation in HOQ can be regarded as an assignment minimum flow type problem, where we seek to minimize the costs. This approach can be represented as networks, where the nodes are the requirements and development parameters, and the edges represent the relationship between them considering the capacity constraints.
2) Hypergraph-based representation and analysis of the HOQ
  In an ordinary graph, an edge connects exactly two nodes so only pairwise interactions can be analyzed, while a hypergraph is a generalization of a graph in which a hyperedge (development parameters) can join any number of vertices (customer requirements), form clusters and allows to analyze group interactions. The generalization of hypergraphs allows hyperedges to become vertices [10], which means that we can evaluate the connection between hyperedges at the same time. Therefore, in the case of QFD, the correlation matrix of development parameters can jointly be analyzed with the relationship matrix. Furthermore, the dual hypergraph represents the swapped roles of vertices and hyperedges. So vertices will define development parameters, and the hyperedges will be the customer requirements. It provides further information about the importance of the elements; therefore, focus areas and possible investment areas can be better identified. Network tools such as centrality measures support identifying the importance of development parameters and where to allocate resources. Furthermore, hypergraphs can be weighted, and partitioning can be made based on constraints.

We believe that the proposed aspects by directly including the correlation matrix in importance ranking and revealing cross-relationships as well as indirect interactions are addition to previous QFD research and promote more efficient product planning and development strategy-making.

In Section 2 a systematic overview of QFD-related research is presented. Then, in Section 3 the network-flow and hypergraph-based methods of improving QFD method are introduced, while their applicability and perspectives on strategic planning are presented through an application study in developing sustainable design of costumer electronic products with minimal impact on environment.

## 2 Overview of the QFD method and related developments

This section provides an overview of the utilization and extension of QFD and highlights the research gap. In order to enhance the precision and accuracy of the outcomes of QFD, this study promotes network-flow and hypergraph-based solutions.

**Figure 1:**
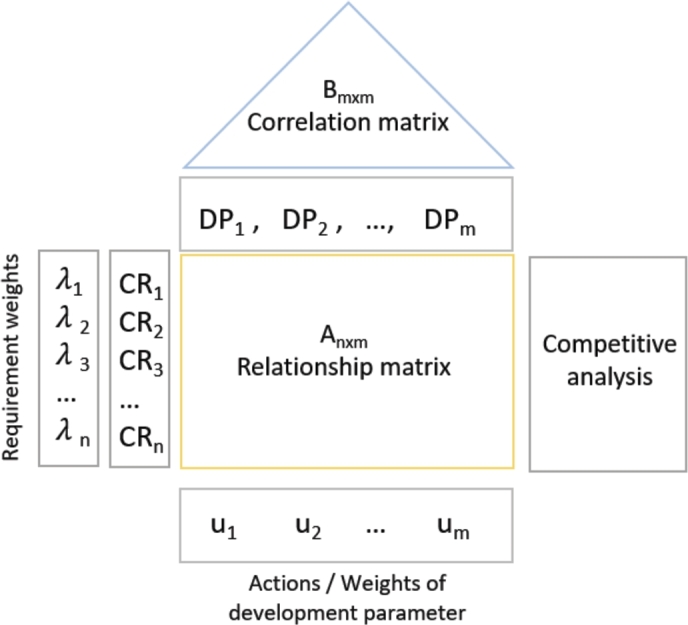
Structure of the House of Quality with variables indicated.

QFD was developed in the 1960's and the related publications have significantly increased, spread in several fields and promote a multidisciplinary viewpoint. The QFD related processes, purposes, users, and tools are described in an ISO 16355 standard [5]. The basic structure of the QFD is formalized and its schematic visualization is shown in Fig. 1.

The original QFD is built as follows:

• First, the customer requirements are gathered and weighted according to the customers' voice. Let *CR* be a set of customer requirements. Each $CR_{i}$ is weighted with $\lambda_{i}$, i=1,...,n.
• Then, these requirements are turned into development parameters *DP*, where $DP_{j},j=1,...,m$ is the *j*-th development parameter.
• The relation of the customer requirements and development parameters form an n×m matrix *A*, which relationship matrix is filled according to the strength of the relationship between them (e.g.: strong (5), medium (3), low (1), non (0)). The value of $a_{i,j}\;(i\in CR,j\in DP)$ indicates the strength of the relationship between the *i*-th requirement and *j*-th development parameter. $a_{i,j}$ can also be considered as an efficiency parameter that defines how important is it to fulfill the given $DP_{j}$ development parameter to meet $CR_{i}$ customer requirement.
• Then, the features of the developing product are compared with similar competitor products on the market, so a strategic plan to position the product is made.
• The relationship of the defined development parameters is explored in a symmetric m×m *B* matrix, where $b_{j,k}$ highlights how would the change in parameter *j* effect parameter *k*. Note, that $b_{j,k}=b_{k,j}$ and $b_{jj}=0$. The correlation between the development parameters can be positive, negative, or neutral and quantified such as 1, -1, 0 accordingly. The setting of these parameters are based on the expertise of the development team. However, the general QFD considers the correlation matrix more as additional information for the development team still does not calculate with it during the importance ranking process.
• The importance ranking value of the development parameters is indicated with $u_{k},k=1,...,m$ and calculated as: $\sum_{j\in DP}\lambda_{i}a_{i,j}\;\forall i\in CR$.
• Finally, the competitive analysis with similar products on the market supports defining the specific product parameters and market strategy.

The application of the above-introduced general QFD method is widely accepted. However, the related drawbacks initiated its improvement and extension to overcome some of the disadvantages of the general QFD. In the following, we systematically answer questions regarding these drawbacks, application fields, and extension potentials:

*What are the major drawbacks of the general QFD method?*

One of the major drawbacks of the general QFD is that the correlation matrix of design parameters is not directly implemented in the importance ranking process. Therefore, the effect of investments in development parameters on other parameters and customer requirements is not represented clearly. The interaction between development parameters remains unfolded, resulting in worse efficiency in meeting customer requirements. Furthermore, if we have limited capacities, we are unsure which parameters we should develop to best meet customer requirements since the importance ranking may indicate selecting actions (development parameters) that are negatively correlated. The diverse application area of the method has initialized the extension of QFD with new approaches. The extensively improved QFD methods seek to overcome the drawbacks such as the importance value is not clearly stated, negative numbers are not accepted in the analysis, the construction and evaluation can be time-consuming, the prioritization is mainly qualitative and subjective, it is hard to categorize parameters, provides single-layer evaluation [11] and it cannot be used effectively when multi-criteria are required [12]. Furthermore, the general evaluation of QFD considers the correlation matrix of development parameters, and it serves as additional information for the development team. Still, the calculation for identifying the most critical development parameters does not include it directly.

*What are the major application fields of QFD-related methods?*

The QFD method and its extensions has span over in several application fields starting from manufacturing or assembly [13], transportation services [14], risk assessment [15], supply chain selection [16], sustainable [17] and green supply chain [18], healthcare [19], the identification of design factors of circular economy [20] or water system performance [21] have also been studied. Education [22] and its development [23] have also gained increasing focus as educational planning is one of the key priority in high-quality systems in organizations and in education [24]. QFD has been utilized in identifying new business opportunities based on technology-driven technology road maps [25], supporting a multi-objective decision-making model in the cloud platform [26]. Furthermore, a customer requirement-driven service system design and computer-aided design in an industrial context have also been improved by integrating the QFD concept in their analysis [27].

*What methods and approaches have been used to improve QFD and which part of the House of Quality has been utilized in previous researches?*

An extended approach to QFD is the *cascade of houses* or the *waterfall relationship of QFD matrices*, which is an interlocking structure to link ends and means at each stage [28]. It is consisted of four phases: product planning, part deployment, process planning, and production planning. In each phase, the relationships between inputs (WHATs) and outputs (HOWs) are established by a relationship matrix called the house of quality (HOQ). This structure enables “multifunctional perspectives, interpretation, and communication to be fully coordinated along the product-process development cycle” [28].

The joint utilization of *QFD and the Kano model* holds great potential for future researches [29] as allows a better understanding of customer needs and “analyzing the effect of meeting customer needs on customer satisfaction levels” while translating these needs into product development and product design requirements [30]. The Kano model is a method used to decide which attributes are most influential on customer satisfaction regarding product or service development and design [31]. The relationship between the Kano model and the QFD method is used “to translate consumer needs and improve customer service attributes in order to improve product quality” [30]. The integrated Kano model into QFD has been utilized, for example, to evaluate digital library [32], or to develop classroom furniture to reach better ergonomic design while satisfying customers [33]. QFD and Kano have been extended with, e.g., SERVQUAL to examine front office quality of hospital [34]; with fuzzy Kano to classify aesthetic attributes of SUV car profile [35]; Quantitative Kano Model and Fuzzy QFD approach for optimizing design method of express delivery service [36]; with hierarchical decision-making approach for improving e-service of brokerage in Iran [37]. For the QFD optimization approach has been combined with a mixed integer linear programming model and Kano model [38].

These methods consider the whole HOQ; however, they do not insert the correlation matrix of development parameters into their calculation so the cross-relationships and possible interactions between development parameters and their impact on satisfying customer needs are neglected. Another “drawback of this integration is that it increases the steps of translating the voice of the customer into technical parameters. Hence, the lead time of developing the innovative products will be increased, which is intolerable given the intensified competition prevailing in the markets” [39].

The *fuzzy approach of QFD* has been applied in several fields [40], which perspective allows to cope with uncertainties associated with inputs while translating customer voice into engineering specifications [41]. The fuzzy QFD approach has been utilized, for example, in the enhancement of electric vehicles' market competitiveness [42], in risk assessment on multimodal transport network [43], in sustainable design of consumer electronics products [41], or in planning for sustainable stakeholder engagement based on the assessment of conflicting interests in projects by utilizing cascade of houses approach [44]. Furthermore, a method based on picture fuzzy linguistic sets and evaluation based on distance from average solution has been developed to improve the efficiency and effectiveness of QFD in prioritizing engineering characteristics [45]. Optimization methods appear jointly with the application of fuzzy QFD, from which the following researches integrate the correlation matrix into their analysis. Li et al. proposed a systematic risk management framework that combines QFD, fuzzy analytic hierarchy processes (AHP), fuzzy failure mode and effects analysis (FMEA), and nonlinear goal programming for the development of a decision support model for risk management of hazardous materials road transportation and to determine the optimized fulfillment level of each risk measure [46]. A *nonlinear programming*-based fuzzy regression and optimization have been utilized to develop, e.g., a decision model for setting and optimizing target levels in QFD [47]. Furthermore, QFD and fuzzy *linear programming* model have also been introduced, e.g., in the construction industry [48], or for new product planning [49]. Optimization models such as *complexity reduction* of a design problem in QFD using decomposition [50], or for supply chain resilience strategy for large passenger aircraft [51] have been developed. These methods efficiently utilize the interactions between the development parameters but are complex and primarily specialized for a development field, which cannot be entirely generalized.

There are also variations for the joint application of *QFD and (fuzzy) AHP*. QFD-AHP are commonly integrated techniques for decision-making purposes [52]. Their joint application has been utilized in manufacturing, supply chain, higher education, strategy, service, marketing, and energy, as well as sustainability [52]. The main advantage of integrating QFD and AHP is that they can rank choices in the order of their effectiveness in meeting the functional objective, mainly at the conceptual design stage [53]. The combination of QFD, fuzzy AHP, fuzzy FMEA, and nonlinear goal programming for hazardous materials road transportation has been proposed that considers complicated situations in which the risk evaluation information is insufficient or incomplete [46]. Another approach integrates QFD, AHP, and data envelopment analysis (DEA) for effective development of B2B product design and concept selection [54]. Furthermore, QFD and AHP has been implemented for the cascade of houses and used as quality achievement tool in healthcare [55]. A major drawback of the proposed integrated approach is due to AHP, which may be time-consuming in reaching consensus [54]. Furthermore, due to the hierarchical structure of AHP it obtains pairwise comparison between the elements, but not able to identify group interactions. Cost and capacity constraints are not included in the beginning of the research [53] and the integration of AHP into the QFD stops after the first phase [7].

QFD can be represented as a network where the relationship between the matrices can be analyzed and visualized. The tool of network science provides further information about the importance of the elements and bottlenecks of the network [56]. The joint application of *QFD and analytical network process (QFD-ANP)* [57], *artificial neural network (ANN)* [4] have been studied. The joint application of QFD and ANP has been utilized, for example, in e-service strategy formulation [57]. A *zero-one goal programming* methodology has been introduced that also includes the importance levels of development parameters (correlation matrix), which are derived using the “ANP, cost budget, extendibility level, and manufacturability level goals” to determine the development parameters to be considered in designing the product [58]. The network-based approach of QFD regarding CNC machine tool development is proposed and reveals the potential of utilizing the correlation network [11]. The potential in analyzing cascade of houses through networks has been highlighted [56], however the correlation matrix is missing from the computation.

Beyond the pairwise network structure, the potential in representing the HOQ with *hypergraphs* has been briefly introduced in a prior research in product family planning for mass customization [59]. However, the concept is introduced briefly and its comprehensive utilization is not discussed or highlight is additional potential in identifying the indirect potentials and metrics.

*How can hypergraph and network flow-based approaches contribute to the improvement of QFD?*

The overview of the QFD-related literature revealed several improvement methods of QFD in various application fields to overcome its limitations. However the following aspects still lack of attention:

• general integration of the correlation matrix for reproductivity in a wide application area
• consideration of cost and capacity constraints
• analyzing group interactions beyond pairwise comparison of the elements
• visual illustration of the direct and indirect interactions between the elements

Therefore, this study promotes approaches that satisfy the aspects mentioned above and enhance the precision and accuracy of the outcomes of the QFD method. Each method directly integrates the properties of the correlation matrix, so it supports better resource allocation based on the cross-relationships and can be generally applied for QFD and can be further customized for specific application areas if needed. We propose the application of 1) network flow analysis, which is an efficient approach to support optimal resource allocation in QFD and identify investment areas while considering different capacity constraints [9]. The network structure provides a possible visual tool for representing the interconnections and pairwise connections between the elements. In addition to the network-based approach, a 2) hypergraph-based analysis of QFD is proposed to identify group interactions. Hypergraphs are great tools for visualizing interactions of the relationship matrix, the correlation matrix, and their joint consideration. Beyond the representation of the interrelationships, centrality measures can identify the importance of elements. Due to these characteristics, more efficient resource allocation and strategic planning of product development can be supported. The proposed improvement aspects of QFD are discussed and formalized in Section 3.

## 3 Methods for improving the QFD method

**Figure 2:**
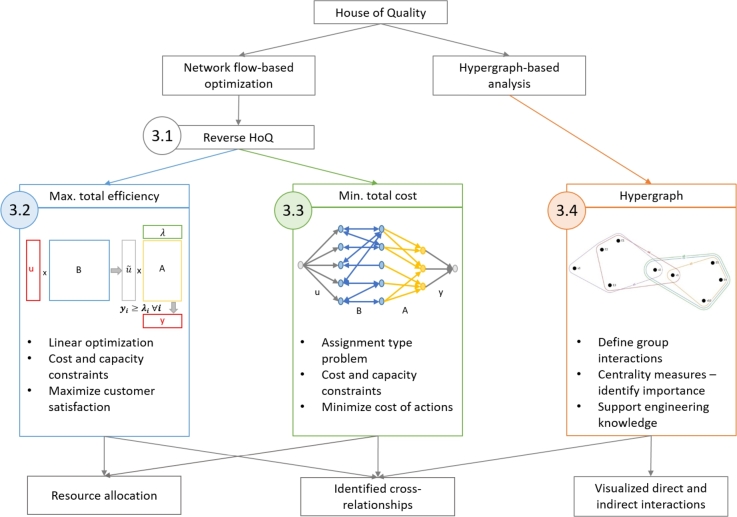
The framework of improving the QFD method with network flow- and hypergraph-based approaches. The methods are labeled according to their subsection.

This section discusses the proposed network flow- and hypergraph-based methods for improving QFD. Fig. 2 represents the framework of the proposed improvement methods and indicates the logical connections between the developments, as well as the structure of their interpretation.

Each method integrates and utilizes the properties of the whole House of Quality, including relationship and correlation matrices. The joint analysis of the relationship and correlation matrices allows for identifying the cross-relationships between the elements. Therefore, better resource allocation and strategic planning can be made even while considering cost and capacity constraints. The proposed methods can be utilized under different development strategies due to their specific objectives.

### 3.1 Formulation of the house of quality

**Figure 3:**
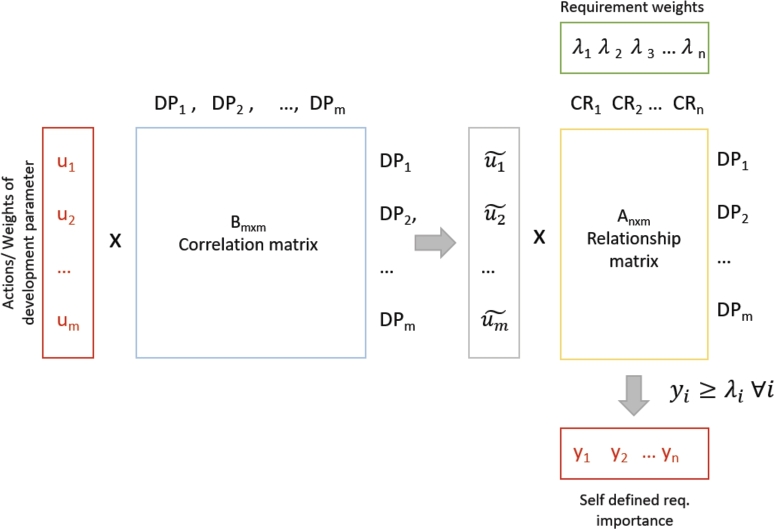
Reverse approach of identifying the importance of actions/development parameters in QFD.

This section presents the mathematical formulation of QFD as a linear optimization problem. Linear programming is a widely accepted operations research tool in practice. It is common practice to assume that complex systems are linear within given limits, and even the most general cost functions are fixed and consist of proportional elements. These assumptions are only valid to a certain extent, but in general, the inaccuracy and uncertainty are not to such an extent that affects selection decisions. The proposed aspect is not a simple linear optimization. It involves a reverse approach to the HOQ with the general idea of optimizing the importance ranking of development parameters under possible capacity or cost constraints while considering the whole HOQ, including the interactions between development parameters. The uncertainty-aware optimization is now out of the article's scope but promotes a possible future research direction. The structure of considering QFD as a linear programming problem is shown in Fig. 3.

Previously we defined $u_{k}$ as the weight of development parameters/actions. Let consider now $u_{k}$ as a decision variable formed in Equation (1):

(1) $$u_{k}\in \{0,1\}\;\forall k=1,...,m,$$

where $u_{k}=1$ if we select (invest in) development parameter *k*, 0 otherwise.

The correlation matrix of the selected development parameters formed as follows in Equation (2):

(2) $$\overset{˜}{u_{k}}=\sum_{k=1}^{m}b_{j,k}u_{k}\;\forall j=1,...,m$$

where $\overset{˜}{u_{k}}$, k=1,...,m holds the properties of development parameters' relationship. It defines how much $u_{k}$ is interconnected with other development parameters and how much it can effect customer requirements.

Then, $A\overset{˜}{u}$ gives the relationship matrix including the effect of correlations between development parameters, where the values show the strengths of relationship between customer requirements and development parameters. The column sum of customer requirements returns *y*, where $y_{i},i=1,...,n$ refers to the self defined requirement importance value of the *i*-th customer requirement, as shown in Equation (3):

(3) $$y_{i}=\sum_{i=1}^{n}a_{i,k}\overset{˜}{u_{k}}\;\forall k=1,...,m$$

$y_{i}$ refers to the efficiency of satisfying the *i*-th customer requirement. It can also be considered the benefit of the investments in the selected development parameters. The higher its value, the more effectively the development parameters have been chosen to meet customer requirements. It holds the properties of both the relationship matrix and the correlation matrix. Therefore, beyond the direct interaction between customer requirements and actions (development parameters) are considered, the indirect connection of other actions and customer requirements is involved by integrating the correlation between development parameters.

To support efficient resource allocation in product development projects, we have to optimize the selection of development actions. Hence, we know how the investment in different development parameters would satisfy customer requirements. We can define two objectives related to QFD that satisfy customer requirements under possible constraints:

• maximizing the efficiency of the actions
• minimizing the total cost of actions

The former objective is formulated in Section 3.2, while the latter is discussed in Section 3.3.

### 3.2 Maximizing the efficiency of actions

We often have to face limited resources in product development projects, e.g., finite budget, which has to be allocated to a limited number of development projects/development parameters, limited inventory or labor work, etc. Each development parameter has a unite $c_{k}$ cost, and we have a finite capacity *C* that we can allocate to/invest in the development parameters. Furthermore, we can have a maximum *M* limit of how many development parameters we can focus on with the same capacity constraint.

Therefore, as indicated in Equation (4) the sum of each action's cost should not exceed capacity *C*:

(4) $$\sum_{k=1}^{m}c_{k}u_{k}\leq C,$$

where $u_{k}\in \{0,1\}\forall k=1,..,m$ is a decision variable that takes value of 1 if the *k*-th action is selected, 0 otherwise. The number of selected actions should not exceed the *M* constraint: $\sum_{k=1}^{m}u_{k}\leq M$.

By selecting *M* development parameters, we want to know how beneficial our investment is. Equation (5) indicates the objective, which is to optimize the selection of development parameters so the efficiency of the selected activities is maximized:

(5) $$max\sum_{i=1}^{n}y_{i},$$

where $y_{i}$ will refer to the benefit - the satisfaction level of customer requirements, and each self-defined benefit has to meet each customer requirement, as shown in Equation (6):

(6) $$y_{i}\geq \lambda_{i}\;\forall i=1,..,n$$

The defined requirement importance value of the *i*-th customer requirement must be greater than or equal to the *i*-th customer requirement weight. It means that the selected development parameters benefit and customer requirements are satisfied, and still, the maximum capacity is not exceeded.

Improving QFD by optimizing the selection of development parameters by maximizing the efficiency of cross-relationships under capacity and cost constraints can be a useful technique in development projects where customer satisfaction is the highest priority, but we have to work with a finite capacity.

### 3.3 Assignment as minimum cost flow problem

QFD can be considered as an assignment type problem, where we have to decide how much and which action we invest in. This can be formulated as a linear programming problem and also represented as a network flow problem, where the nodes are the development parameters and the customer requirements, and the edges represent the connections between them. The edges have costs and capacities, and the goal is to achieve a given amount of flow with the minimum cost possible.

Let's assume we have a group of development parameters (actions) to satisfy a set of customer requirements. There is a cost for assigning an action to a requirement for each action and requirement. The problem is to assign each action to at most one task, with no two actions performing the same requirement while minimizing the total cost. This problem can be visualized as a graph, where the actions and requirements are the nodes, while the edges represent the possible ways to assign them to each other. Each edge has a unit cost.

**Figure 4:**
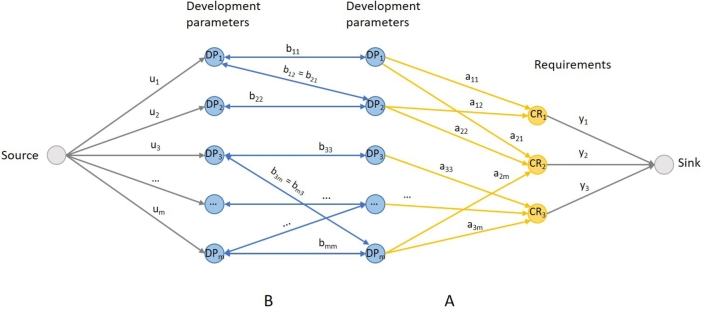
Schematic representation of QFD as a bipartite graph. The blue nodes are the development parameters, and the yellow nodes represent the customer requirements. The arrows show the relationship between nodes, the blue ones refer to matrix B, and the yellow is matrix A.

The schematic representation of QFD as a network flow is shown in Fig. 4, where the nodes are the requirements and the development parameters, while the edges represent the relationship between them. The relationship matrix is represented as the edge connection between node $CR_{i}$ to node $DP_{j}$. The edges between $DP_{j}$ and $DP_{k}$ shows the correlation matrix of development parameters.

Equation (7) indicates that the decision variable is the weight of the development parameter, which takes 1 if the development parameter is selected, 0 otherwise:

(7) $$u_{k}\in \{0,1\}\;\forall k=1,...,m$$

Each edge has a unit cost $c_{k}$ for executing an activity. The problem is to find a flow with the least total cost.

The objective is therefore to minimize the total cost, as described in Equation (8):

(8) $$min\sum_{j=1}^{m}c_{k}u_{k}$$

This flow method can be customized and set more easily by setting capacity and cost constraints to the model. This aspect is favorable in a highly cost-oriented environment, however it may dismiss other important parameters due to the minimum cost focus. Furthermore, the assignment as minimum cost flow method considers only pairwise interactions and group interactions may remain uncovered.

### 3.4 Hypergraph-based representation of QFD

Hypergraph theory is a dynamically developing field in operation research [60]. Hypergraph has “emerged as a better tool for modeling group interaction” compared to simple graph representation [61], which feature is crucial in analyzing QFD. Hypergraphs have been proved to be efficient in representing interacting elements and supporting design of interactions [62]. The traditional network concept defines the pairwise interactions within nodes, however, a hypergraph is a generalization of a graph in which an edge can join any number of vertices and forms clusters. Such as, in QFD, there is more than one development parameter connected to one customer requirement, which can form a cluster that defines what actions are needed to satisfy the requirement. It works inversely, as one development parameter can be connected to more customer requirements, which cluster defines which requirements can be improved by the given development parameter.

#### 3.4.1 Formation of hypergraphs

A hypergraph is a generalization of a graph in which an edge can join any number of vertices. In contrast, in an ordinary graph, where an edge connects exactly two vertices.

A hypergraph can be represented by the incidence matrix H=(V,E). In this structure, $V={\left\{v_{i}\right\}}_{i=1}^{n}$ is a set of vertices, and $E={\left\{e_{j}\right\}}_{j=1}^{m}$ indicates a family of hyperedges where each $e_{j}$ is a subset of *V* [10].

Table 1 summarize the characteristics of the vertices that could be defined in the case of QFD. A vertex can be a customer requirement, which has different development parameters needed for its satisfaction. A sub-group of this vertex is another customer requirement that involves at least those development parameters as the former. The other perspective is when a vertex is a development parameter that satisfies customer requirements. Its sub-groups are those development parameters that satisfy at least those requirements as the former.

**Table 1:** Vertices and their characteristics in QFD.

	Vertices	
	Customer requirements	Development parameters
Properties	Development parameter required for its satisfaction	Customer requirement effected
Aggregation	Based on property similarity	Based on property similarity
Corresponding sub-groups	Customer requirements, that requires same development parameters for its fulfillment	Development parameters, that correspond to the fulfillment of the same customer requirement

Let's consider a hypergraph, where vertices are the customer requirements (*CR*) and hyperedges are the development parameters (*DP*).

The size of a hyperedge is indicated with $\left|e_{j}\right|$, that shows how many vertices are part of the set. Hyperedges' size can differ and take only a single vertex {v}⊆V or can take the whole vertex set *V* [63]. For example, how many customer requirement involves the same development parameter (hyperedge) for its fulfillment. In case a hyperedge involves exactly two vertices ($e=\{v_{1},v_{2}\}$ so its size |e|=2), then it represents the same as a graph edge. These graphs are identified as being “2-uniform” hypergraphs [63].

Each hypergraph has a unique Boolean incidence matrix. Considering hypergraph *H*, it has an $F_{n\times m}$ incidence matrix, where [64]:

(9) $$f_{i,j}=\left\{ \begin{array}{cc} 1, & \text{if }v_{i}\in e_{j}\\ 0, & \text{otherwise } \end{array}\right.$$

Based on Equation (9), this incidence matrix will represent the relationship matrix of customer requirements and development parameters. It will be 1 if there is any relationship between the customer requirement and the development parameter, 0 otherwise.

Let's consider a QFD as a hypergraph with the following elements:

• The set of vertices is the customer requirements:
  $V=\{v_{1},v_{2},v_{3},v_{4},v_{10}\}$.
• The family of hyperedges ${\left(e_{j}\right)}_{j\in \{1,2,...k\}}$, where $\left(e_{j}\right)$ is the subset of customer requirements, which are involved in the *j*-th development parameter (DP)

The incidence matrix of *H* example hypergraph is shown in Table 2. The edges represent the development parameters from $DP_{1}$ to $DP_{5}$, and the vertices are the customer requirements $CR_{1},CR_{2},CR_{3}$ and $CR_{10}$.

**Table 2:** Incidence matrix of hypergraph *H*.

v1v2v3v10e1(1101)e21001e31101e41100e50110

The incidence matrix of the hypergraph shows the connection between *CR* and *DP*. A hyperedge $e_{j}$ is said to be incident with a vertex $v_{i}$ when $v_{i}\in e_{j}$. For example, if development parameter: $e_{5}$ is a hyperedge that refers to $DP_{5}$ parameter, it involves the vertices of $v_{2},v_{3}$ customer requirements, but not $v_{1},v_{10}$. So we can say, that $e_{5}$ development parameter contributes to the fulfillment of $v_{2},v_{3}$ customer requirements, but has no direct effect on $v_{1},v_{10}$.

**Figure 5:**
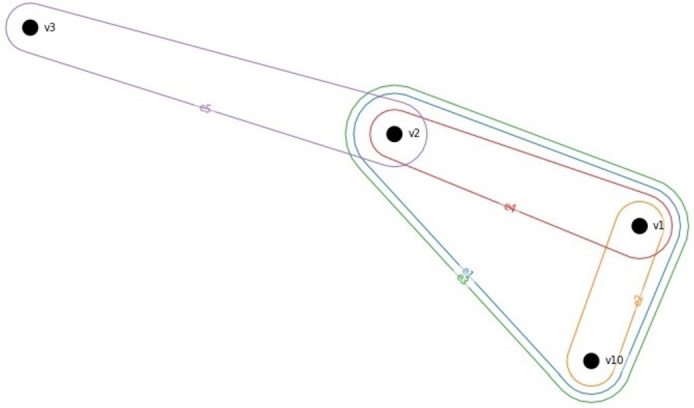
Visual representation of hypergraph *H*.

The visual representation of the incidence matrix is shown in Fig. 5. It clearly indicates the interactions between the elements. For example $e_{5}$ and $e_{4}$ joins the same vertex $v_{2}$ as both development parameters influence customer requirement $v_{2}$; or $e_{1}$ and $e_{3}$ involves exactly the same vertices and they tend to be analog each other if we only consider the incidence matrix without weights.

The degree of a vertex (d(v)) is the number of hyperedges it connects to, and represented as d(v)=|{e:v∈e}| [65].

In this case, the degree of $v_{1}$ customer requirement is $d(v_{1})=4$ as it is connected to development parameters $e_{1},e_{2},e_{3},e_{4}$. It shows how many development parameters is a customer requirement directly linked to and how central it is.

An interesting approach is the fuzzy hypergraph $\overset{˜}{H}$ as $\overset{˜}{H}=(\nu ,\varepsilon )$, with $\nu ={\left\{\nu_{i}\right\}}_{i=1}^{n}$ a set of vertices, and $\varepsilon ={\left\{\overset{˜}{E_{j}}\right\}}_{j=1}^{m}$ a family of hyperedges with each $\varepsilon_{j}\subseteq \nu$ [66]. The hyperedges $\overset{˜}{E_{j}}$ will be a fuzzy set of vertices. Then the degree of participation of vertex, $v_{i}$ to edge $\overset{˜}{E_{j}}$ is defined by $\mu_{i}(v_{i})$. The fuzzy hypergraph can also be represented with its fuzzy incidence matrix, where the element $f_{ij}$ represents the membership degree of $v_{i}$ to $\overset{˜}{E_{j}}$ [66].

Since fuzzy QFD is a widely acknowledged concept, the idea of fuzzy hypergraph could provide additional value to its representation and validation.

The incidence matrix of fuzzy hypergraph $\overset{˜}{H}$ is indicated in Table 3. The {0,1} values are replaced with the data that represents the strength of relationship between the elements (0*- weak, 0.5 - medium, 1 - strong).

**Table 3:** Incidence matrix of a fuzzy hypergraph $\overset{˜}{H}$.

v1v2v3v10e1(0.50.501)e20.5000⁎e30⁎0.500.5e40.5100e5010.50

A fuzzy hypergraph $\overset{˜}{H}$ can be cut at *α* level, so then we obtain the *α*-cut hypergraph $\overset{˜}{H_{\alpha }}=(\varepsilon_{\alpha },\nu_{\alpha })$ [66]. If α=0.8, we keep only values equals or above *α*. Then, we get $H_{0.8}$, which incidence matrix only contains those verticies that meet the requirement.

The example fuzzy hypergraph cut at α=0.8 is represented in Table 4. Then, in QFD, only the strong relationship values will remain in the relationship matrix.

**Table 4:** Incidence matrix of fuzzy hypergraph $\overset{˜}{H}$ cut at *α* = 0.8 level.

v1v2v3v10e1(0001)e20000e30000e40100e50100

We can filter the relationship matrix and select only the lowest and the highest values represents their connection.

A weighted hypergraph is denoted as: H=(V;E;w). $w_{i,j}$ can be considered as the hyperedge weight of the relationship between *i*-th requirement and *j*-th development parameter. A hyperedge without a weight attribute has a default value of 1. Considering QFD, we can set weights according to AB=W, which provides additional information and defines the effect and importance of each development parameter on customer requirements.

Furthermore, a hypergraph that swap the roles of vertices and hyperedges is called a dual hypergraph. The dual hypergraph of *H* is represented as $H^{⁎}=(V^{⁎},E^{⁎})$. This structure includes a vertex set $E^{⁎}={\left\{e_{j}^{⁎}\right\}}_{j=1}^{m}$, and $V^{⁎}={\left\{v_{i}^{⁎}\right\}}_{i=1}^{n}$, which is the family of hyperedges where $v_{i}^{⁎}:=\{e_{j}^{⁎}:v_{i}\in e_{j}\}$. The incidence matrix of a dual hypergraph is transposed, denoted as $F^{T}$. Furthermore, the dual of a dual hypergraph will result in the original hypergraph, ${\left(H^{⁎}\right)}^{⁎}=H$ [64]. This dual graph approach allows to model QFD, where vertices are customer requirements, and hyperedges are the development parameters as well as when vertices are development parameters, and hyperedges are the customer requirements. It allows better identification of importance and focus areas, therefore more efficient resource allocation.

To better identify and visualize the relationship between development parameters, the correlation matrix of development parameters can also be represented in an incidence matrix indicated in Table 6. Since the correlation between development parameters also differs, we can make it into a fuzzy incidence matrix, as shown in Table 5.

**Table 5:** Incidence matrix of the correlation matrix.

e1e2e3e4e5e1(00011)e200011e300011e411100e511100

**Table 6:** Fuzzy incidence matrix of the correlation matrix.

e1e2e3e4e5e1(0000.30.3)e20000.30.3e30000.30.3e40.30.30.300e50.30.30.300

**Figure 6:**
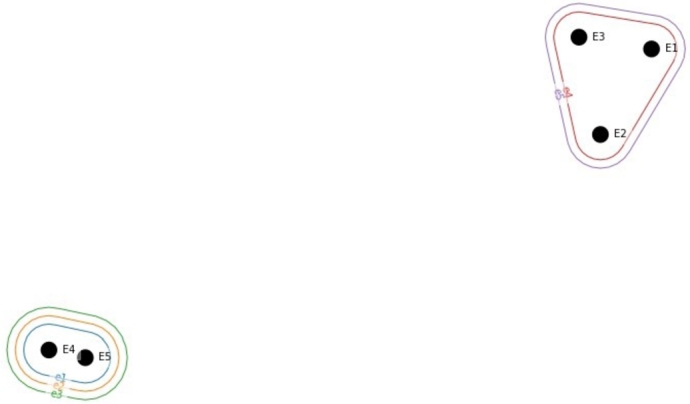
Hypergraph representation of the correlation matrix.

Then, the relationship between the development parameters can be visualized as shown in Fig. 6. The vertices and edges are the development parameters. Vertices $\left\{E1,E2,E3,E4,E5\}=\{e_{1},e_{2},e_{3},e_{4},e_{5}\right\}$. It nicely represents which parameters affect each other. In this example, there is only a positive correlation (0.3), but the different directions of the effect could be represented with different colors indicating strong negative, negative, positive, or strong positive relationships.

For the joint analysis of the relationship and the correlation matrix, a generalization of hypergraph can be made, which allows hyperedges to become vertices. Therefore, a “hyperedge *e* may not only contain vertices but may also contain hyperedges, which will be supposed different from *e*” [10]. Therefore, it is possible to visualize the relationship matrix and correlation matrix of the House of Quality jointly.

As an example, $V=\{v_{1},v_{2},v_{3},v_{10}\}$, and $E=\{e_{1}=\{v_{1},v_{2},v_{10},e_{4},e_{5}\}$; $e_{2}=\{v_{1},v_{10},e_{4},e_{5}\}$; $e_{3}=\{v_{1},v_{2},v_{10},e_{4},e_{5}\}$; $e_{4}=\{v_{1},v_{2},v_{10},e_{1},e_{2},e_{3}\}$; $e_{5}=\{v_{2},v_{3},v_{10},e_{1},e_{2},e_{3}\}\}$. An incidence matrix of the above mentioned example is shown in Table 7. The size of the incidence matrix will be |E|×(|V|+|E|).

**Table 7:** Incidence matrix of a generalized hypergraph, where hyperedges become vertices.

v1v2v3v10e1e2e3e4e5e1(110100011)e2100100011e3110100011e4110011100e5011011100

**Figure 7:**
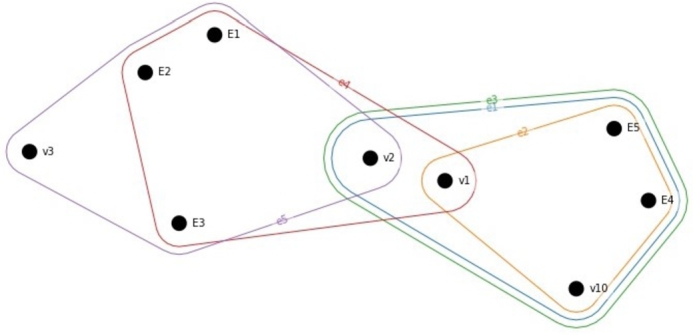
Generalized hypergraph representing the direct and indirect connection of customer requirements and development parameters. Note, that {*E*1,*E*2,*E*3,*E*4,*E*5}={*e*_1_,*e*_2_,*e*_3_,*e*_4_,*e*_5_}.

The visual representation of the generalized hypergraph is shown in Fig. 7. Note that in Fig. 7 the edges $\left\{e_{1},e_{2},e_{3},e_{4},e_{5}\right\}$ are involved as vertices {E1,E2,E3,E4,E5}. Translating to this for QFD, the incidence matrix will include not just the relationship matrix attributes but also the interaction of hyperedges (correlation of development parameters).

It nicely visualizes the interconnections between the elements. For example, $v_{10}$ requirement is directly determined by $e_{1},e_{2},e_{3}$, but due to the correlation between the development parameters, $e_{4}$ and $e_{5}$ has also indirectly affects the efficiency of satisfying this customer requirement.

#### 3.4.2 Hypergraph-based metrics of analyzing QFD

The tools of network science are utilized by defining centrality measures that identify the importance of vertices in a network. Three hypergraph-specific metrics can be distinguished, s-betweenness centrality, s-closeness centrality, and modularity [63]. The closeness centrality indicates how close a vertex is to all other vertices in the network. The betweenness centrality shows the amount of influence a vertex has over the flow of information in the hypergraph.

The meaning of these metrics considering QFD is shown in Table 8.

**Table 8:** Hypernetwork measures and their application in QFD analysis.

Centraliry metric	Application in QFD
s-betweenness centrality	Shows how determinative (important) is the element
s-closeness centrality	Shows how much the element effects other elements
Partition of the hypergraph	Shows how modular is the development project

Hypergraph centrality measures are based on concepts of a hypergraph walk and distance [65]. A hypergraph walk is characterized as an “s-walk”, where *s* indicates the minimum pairwise intersections between adjacent edges. Let consider two hyperedges e,f∈E with an s-walk of length *k*. It can be denoted as a sequence of hyperedges $e_{0},e_{1},...,e_{k}$, where $e_{0}=e,e_{k}=f$ and $s\leq |e_{j}\cap e_{j+1}|$ for all 0≤j≤k−1 [65]. So *s* defines at least how many customer requirement is satisfied by at least two neighboring edges (development parameters). The s-distance is the length of the shortest s-walk between two edges e,f∈E defined as $d_{s}(e,f)$, where s>0. This will be the minimum number of customer requirement satisfied jointly by at least two neighboring edges. In case there is no s-walk between two edges, the s-distance will be infinite [65]. We define two edges s-adjacent, if |e∩f|≥s for s≥1 [67].

If *e* is an edge, then based on [63], the s-betweenness centrality $BC_{s}(e)$ of e is denoted as follows in Equation (10):

(10) $$BC_{s}(e):=\sum_{f\neq e\neq g\in E}\frac{\sigma_{fg}^{s}(e)}{\sigma_{fg}^{s}}$$

where $\sigma_{fg}^{s}$ denotes the number of shortest s-walks from edge *f* to edge *g*. The number of those shortest s-walks that contain edge *e* are represented by $\sigma_{fg}^{s}(e)$. If *e* has high s-betweenness centrality, it means that it intersects with many edges, thereby it has a central role in the model. It can represent the importance of the elements (development parameters).

The s-closeness centrality CC(e) is computed on each of the connected components and denoted as follows in Equation (11), based on [63]:

(11) $$CC_{s}(e)=\frac{|E_{s}|-1}{\sum_{f\in E_{s}}d(e,f)},$$

where $E_{s}=\{e\in E:|e|\geq s\}$.

The harmonic s-closeness centrality $HCC_{s}(e)$ of a hyperedge *e* based on [63], is calculated as the reciprocal of the harmonic mean of distances from *e*, as shown in Equation (12):

(12) $$HCC_{s}(e):=\frac{1}{|E_{s}|-1}\sum_{f\in E_{s},f\neq e}\frac{1}{d_{s}(e,f)}\;.$$

Another possibility to better identify connections, partitioning, can be used, which defines, e.g., two clusters where “the connection among the vertices in the same cluster is dense while the connection between two clusters is sparse” [68].

For a vertex “subset S⊂V, let $S^{c}$ denote the compliment of *S*. A cut of a hypergraph H=(V,E,w) is a partition of *V* into two parts *S* and $S^{c}$. We say that a hyperedge *e* is cut if it is incident with the vertices in *S* and $S^{c}$ simultaneously” [68]. Partitioning defines how connected are the components. If there is a low connectivity between the elements, it can be grouped, which means that the development of these two groups is critical and should focus on separately.

These metrics can be utilized in case of QFD and product development project:

• If a development parameter has a high centrality, then it has a significant effect on other elements in the network, therefore, it can be useful to develop multiple requirements by the investment of this parameter. For example, in case of s=1, in the example QFD, $e_{1},e_{3},e_{4}$ has the highest closeness centrality value 1 and their betweenness centrality equals to 0.33. At s=2, only $e_{1},e_{3}$ remains the most central, and the value of $e_{4}$ is decreasing as it connects only two vertices, while the other three.
• If a customer requirement has a high centrality, then it is influenced by many parameters, therefore, it can be critical to satisfy entirely.
• If the partitioning defines two clusters, then the connection between the two groups is low, therefore, the development of these two groups is critical and should focus on separately.

## 4 Application study on sustainable design of customer electronics products

In this section, the proposed methods introduced in Section 3 are applied for a case study based on a QFD analysis for the sustainable design of consumer electronics products. The input data is adapted from the work of Vinodh et al. [41].

The example study has high importance as “eco-design of electronic products is driven by legislation, and other policy instruments, rising resource prices for limited resources, and consumer demand and market opportunities” [69]. Designers and product developers need to develop electronics products with minimal impact on the environment and beneficial for society and the economy. Sustainable design is based, e.g., on using sustainable materials, minimizing energy usage, or can be recycled at the end of their life cycle. Therefore, for performing more efficient eco-design, the analysis of interactions between requirements from both customer and technical implementation is highly needed.

### 4.1 Introduction of the case study

The original data relies on the work of Vinodh et al. [41], which applies a fuzzy approach and identifies membership functions to describe the connection between elements. For a more straightforward interpretation, now we use only the middle value of the membership functions, where the function takes the highest value.

**Figure 8:**
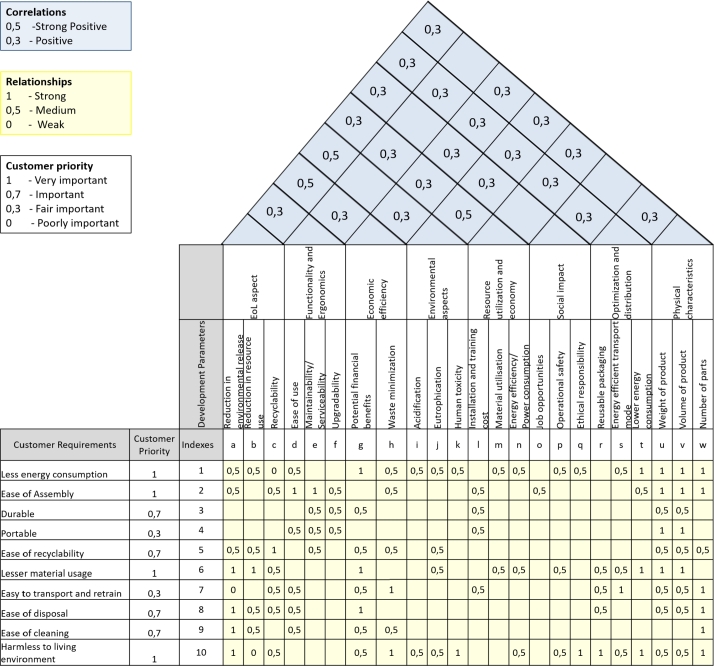
House of Quality for sustainable design of customer electronics products based on Ref. [41].

The input data structure of the QFD is shown in Fig. 8.

Customers have defined ten requirements for electronic customer products ($CR_{i},i=1,..10$), which are the following: Less energy consumption, Ease of Assembly, Durable, Portable, Ease of recyclability, Lesser material usage, Easy to transport and retrain, Ease of disposal, Ease of cleaning, and Harmless to the living environment.

Parameters have been identified influencing the sustainable design of customer electronic products, and the 23 development parameter ($DP_{j},j=1,..,23$) has been categorized into eight dimensions:

• End of Life (EoL) aspects: Reduction in environmental release, Reduction in resource use, Recyclability
• Functionality and ergonomics: Ease of use, Maintainability/Serviceability, Upgradability
• Economic efficiency: Potential financial benefits, Waste minimization
• Environmental aspects: Acidification, Eutrophication, Human toxicity
• Resource utilization and economy: Installation and training cost, Material utilization, Energy efficiency/Power consumption
• Social impact: Job opportunities, Operational safety, Ethical responsibility
• Optimization and distribution: Reusable packaging, Energy efficient transport mode, Lower energy consumption
• Physical characteristics: Weight of product Volume of product, and Number of parts

In the relationship matrix, three levels of interaction are considered: Strong - 1, Medium - 0.5, and Weak -0. The correlation matrix also includes fuzzy values, where only positive - 0.3 and strong positive - 0.5 correlations have been identified between the development parameters.

The original study concluded that the highest importance values regarded the EoL aspect: ‘Reduction in environmental release’, ‘Reduction in resource use’, and ‘Recyclability’ development parameters, followed by economic efficiency: ‘Potential financial benefits’, and ‘Waste minimization’.

However, the case study does not consider capacity constraints, and the correlation matrix of the development parameters is not included in the importance ranking process. In the following Section 4.2, the three proposed methods seek to overcome the limitations mentioned above.

### 4.2 Application of the proposed methods

This section discusses each method's characteristics and compares their results performed on the case study introduced. The correlation matrix of the development parameters is integrated into the calculations, and capacity constraints are defined. Let's assume that we have a finite capacity *C*, which defines how much we can invest in the development projects. Furthermore, assume that we can only focus on ten development actions *M*, so we have to allocate this amount of budget for maximum *M* development parameters. Each development parameter has a unite cost.

#### 4.2.1 Maximizing the efficiency of actions in sustainable electronic device development

The application of linear programming-based QFD aims to maximize its effectiveness based on the connection between the relationship and correlation matrix. It considers capacity constraints and can be utilized when we want to maximize the fulfillment of customer requirements under a finite, e.g., budget constraint. The results of this method are summarized in Table 9 and reflect on the most critical development parameters to satisfy customer requirements.

**Table 9:** Results of applying linear programming for maximizing the efficiency of actions with different settings.

Customer requirements satisfaction level	Capacity constraint (C)	Max. no of development actions selection (M)	Actual selected development actions	Actual efficiency value	Actual cost
Minimum (at least)	no	no	5 pcs - d, e, f, i, k	18,095	12
Maximum	no	10	10 pcs - d, e, f, i, j, k, l, m, n, r	33,679	27
Maximum	yes	10	10 pcs - d, e, f, h, i, j, k, l, m, n	33,586	26

At least five development parameters have to be selected to satisfy customer requirements at least at the level they set as the customer requirement weights. These development parameters are: ‘Ease of use’, ‘Maintainability/Serviceability’, ‘Upgradability’, ‘Acidification’, and ‘Human toxicity’. Its total efficiency value is 18.095 with a cost of 12.

Let's assume that we can invest in a maximum of ten development parameters. The optimization method then selects ten development parameters with the highest efficiency in satisfying customer requirements. If the cost constraint is not included, then the following parameters turned out to be the most effective: ‘Ease of use’, ‘Maintainability/Serviceability’, ‘Upgradability’, ‘Acidification’, ‘Eutrophication’, ‘Human toxicity’, ‘Installation and training cost’, ‘Material utilization’, ‘Energy efficiency/Power consumption’, ‘Reusable packaging’. These parameters highlighted the importance of Functionality and Ergonomics, Environmental aspects, Resource utilization, and economy and one element was selected from Optimization and distribution. Its total efficiency value is 33,679, with a total cost of 27.

If we consider the cost constraint that is under this budget, the optimization method changes first the ‘Reusable packing’ to ‘Waste minimization’, then the total efficiency is 33.586 at C=26.

We can conclude that implementing the correlation matrix changes the optimal selection of development action. This example nicely represents that, however, the original research considered the most critical parameters of the EoL aspect, the optimization method did not include them in the selection as there is a strong positive correlation between EoL aspects and Economic efficiency and Environmental aspects.

This method is useful for identifying those parameters that can bring the highest benefit and by setting capacity constraints, and the customer requirements are still satisfied as much as possible.

#### 4.2.2 Minimum cost flow approach in sustainable electronic device development

The assignment as a minimum cost flow approach seeks to minimize the costs of actions. This approach can be useful when investing in development parameters based on a cost-effective strategy.

According to the assignment problem, we render at maximum one development parameter to each customer requirement while minimizing the total cost.

The optimization method selected the development parameters, which have a total efficiency value of 30.809 and a total cost of 18. The following development parameters have been selected by this method: ‘Reduction in environmental release’, ‘Ease of use’, ‘Maintainability/Serviceability’, ‘Upgradability’, ‘Human toxicity’, ‘Material utilization’, ‘Energy efficiency/Power consumption’, ‘Job opportunities’, ‘Operational safety’ and the ‘Weight of product’.

This method is useful for identifying parameters that meet customer requirements with the lowest cost possible.

#### 4.2.3 Hypergraph-based analysis of interactions in sustainable electronic device development

This section represents aspects of improving QFD by including the correlation matrix in the analysis and providing a visual tool for the better identification of interactions between the elements of the House of Quality. Firstly, the relationship and correlation matrices are analyzed separately to identify the most central/important elements of the HOQ. Then, the joint analysis is discussed, which highlights the direct and indirect interconnections between the elements.

Table 10 summarizes the different representations of QFD matrices and the related most central values based on their s-closeness and s-betweenness values. These critical elements can be considered as key factors for the development project. Each representation is discussed below more in details. The joint analysis of correlation and relationship matrix is discussed at Fig. 13.

**Table 10:** Hypergraph-based metrics of the relationship and correlation matrices. The top central/important values are identified based on their s-closeness and s-betweenness values. (CR - customer requirements, DP - development parameters).

Figures	QFD matrix	Vertices	Hyperedges	s-value	s-closeness	s-betweenness	Top central values

Fig. 9	Relationship matrix	CR	DP	1	1	3,776	a,c,d,b,t,u,v,w
				2	1	12,034	u,v

Fig. 10	Relationship matrix (dual)	DP	CR	1	1	0	all equally centered
				2	1	0,286	1,2,3,6,7,8,9,10

Fig. 11	Fuzzy cut relationship matrix (*α* − 0,5)	CR	DP	1	0,929; 0,813; 0,813	22,214; 6,486; 6,486	w,t,a
				2	1; 0,857; 0,857	5,667; 0,667; 0,667	w,t,a

Fig. 12	Correlation matrix	DP	DP	1	1	0	all equally centered
				2	1	0	all equally centered

**Figure 9:**
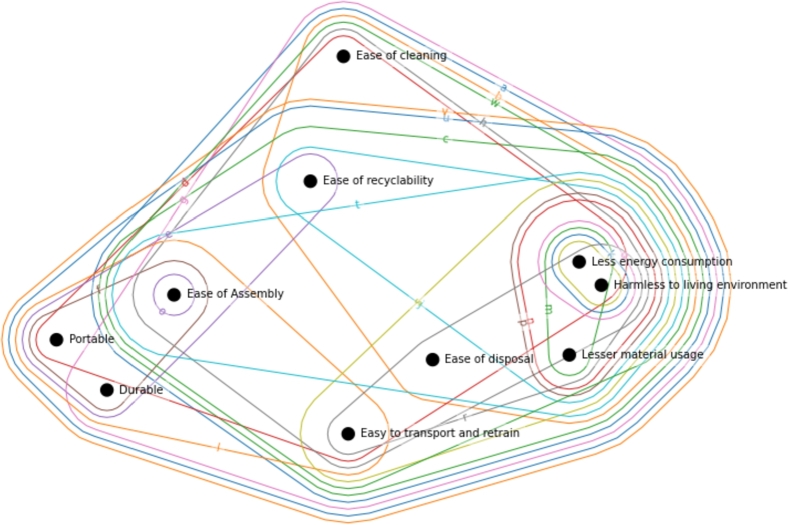
Hypergraph representation of relationship matrix. Vertices indicate the customer requirements, while the hyperedges refer to the development parameters.

**Figure 10:**
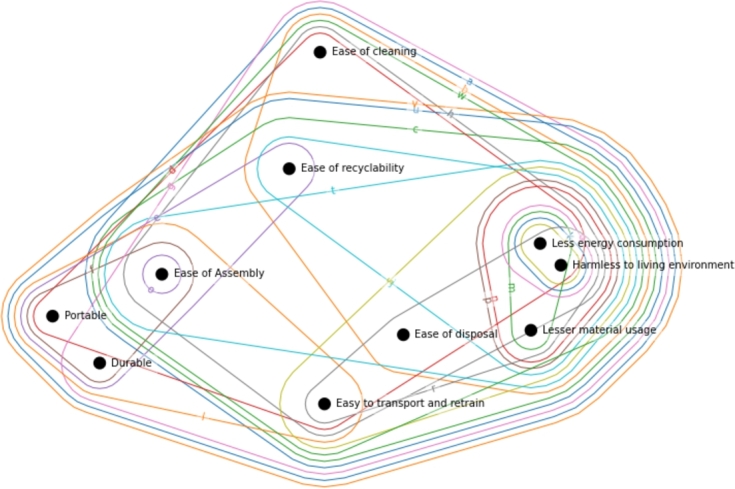
Dual hypergraph representation of the relationship matrix. Vertices indicate the development parameters, while the hyperedges refer to the customer requirements.

The hypergraph-based representation of the relationship matrix is shown in Fig. 9 and its dual hypergraph in Fig. 10. The former defines the hypergraph where verticies are the customer requirements $\left(V=\{CR_{1},CR_{2},...,CR_{n}\}\right)$, and edges are the development parameters $\left(E=\{DP_{1},DP_{2},...DP_{m}\}=\{a,b,...,w\}\right)$. Hyperedges *u* and *v* are the largest as they contain nine out of ten customer requirements vertices, while *o* is the most simple with only one vertex.

Based on s-closeness and s-betweenness metrics, at s=1 the most important and influential development parameters with s-closeness value of 1 and s-betweenness value of 3,776 are a,c,d,h,t,u,v,w - ‘Reduction in environmental release’, ‘Recyclability’, ‘Ease of use’, ‘Waste minimization’, ‘Lower energy consumption’, ‘Weight of product’, ‘Volume of product’. In case of s=2, the most important values remain u,v with s-closeness centrality 1 and s-betweenness value of 12,034.

Fig. 10 indicates the dual hypergraph of the previously defined hypergraph, which has the opposite meaning. The vertices are the development parameters, while edges represent the customer requirements, so it is the transposed form of the previous visualization. For example, hyperedge ‘Portable’ connects the following development parameters: d,e,f,h,l,u,v. It defines which development parameters are needed for satisfying customer requirements. Therefore, the intersections of hyperedges refer to the joint usage of development parameters. The largest hyperedge is ‘Less energy consumption’, containing 18 development parameters. In the case of a dual hypergraph, the s-centrality measures identified that at s=1, each customer requirement is equally central. At s=2, ‘Portable’ and ‘Durable’ requirements finish the second place with s-closeness centrality 1 and s-betweenness value of 0. According to s=3, the ‘Ease of disposal’, ‘Less energy consumption’, ‘Ease of Assembly’, ‘Ease of recyclability’, ‘Easy to transport and retrain’ are determined as the most critical elements with an s-closeness value of 1 and s-betweenness value of 0,676.

**Figure 11:**
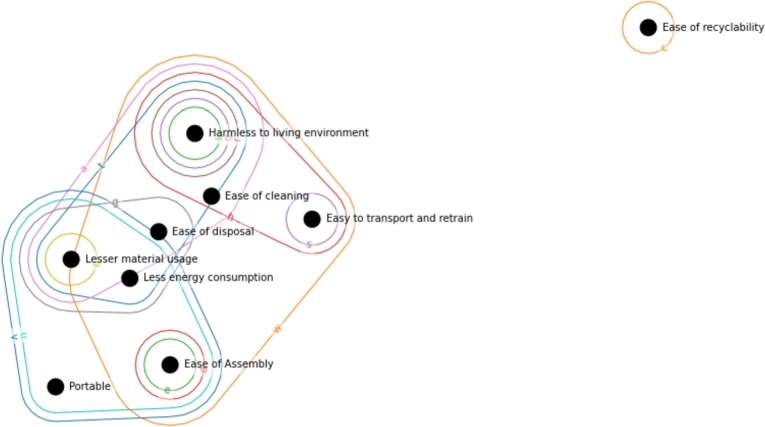
Fuzzy hypergraph cut at *α* = 0.5 level.

Since fuzzy QFD gains increasing attention, we can utilize its properties such as the *α*-cut, where only those values will remain in the incidence matrix above the *α* level. In the case of QFD, it would mean that we cut out elements from the incidence matrix beyond a certain level. Its visual representation is indicated in Fig. 11. So when focusing on areas with the highest relationship values, we set *α* to 0.5, and result in a sparse incidence matrix. Development parameter *w* - ‘Number of parts’ is the largest as it connects six customer requirements, and it became the central element of the hypergraph due to its strong connectivity.

**Figure 12:**
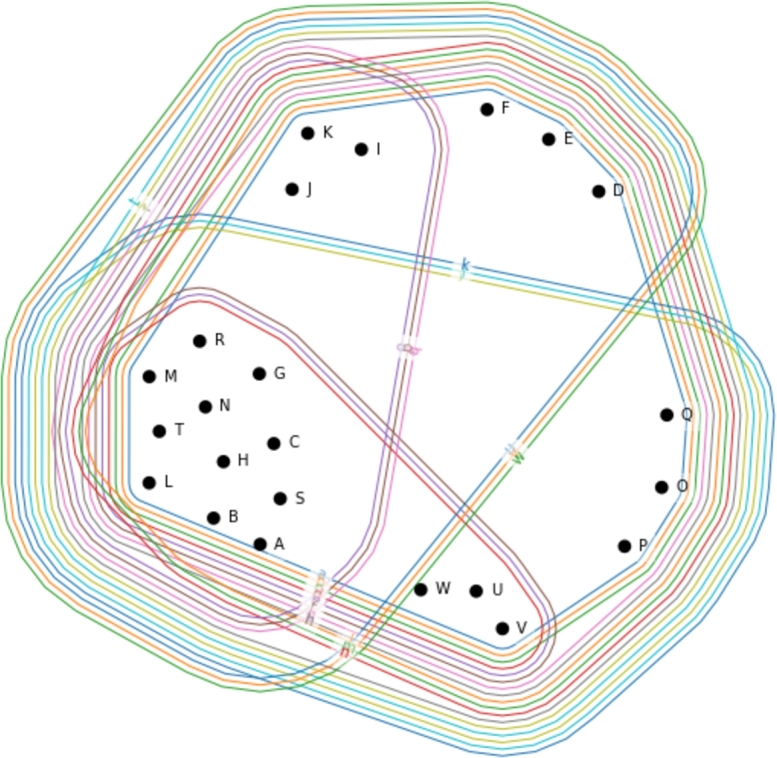
Hypergraph representation of correlation matrix.

The hypergraph of the correlation matrix adds information about how important a development parameter is regarding the interaction between other development parameters, as indicated in Fig. 12. It provides a visualization about the interaction, for example, in the left bottom corner we can see the development parameters d,e,f - ‘Ease of use’, ‘Maintainability/Serviceability’, ‘Upgradability’ correlates with most of the development parameters except p,o,q,k,i,j. Due to the high interconnection between the development parameters, each hyperedge is equally centered. This representation could be further developed by indicating that the direction of interaction could be color-coded. The presented example only consists of positive and strong positive interactions between development parameters.

**Figure 13:**
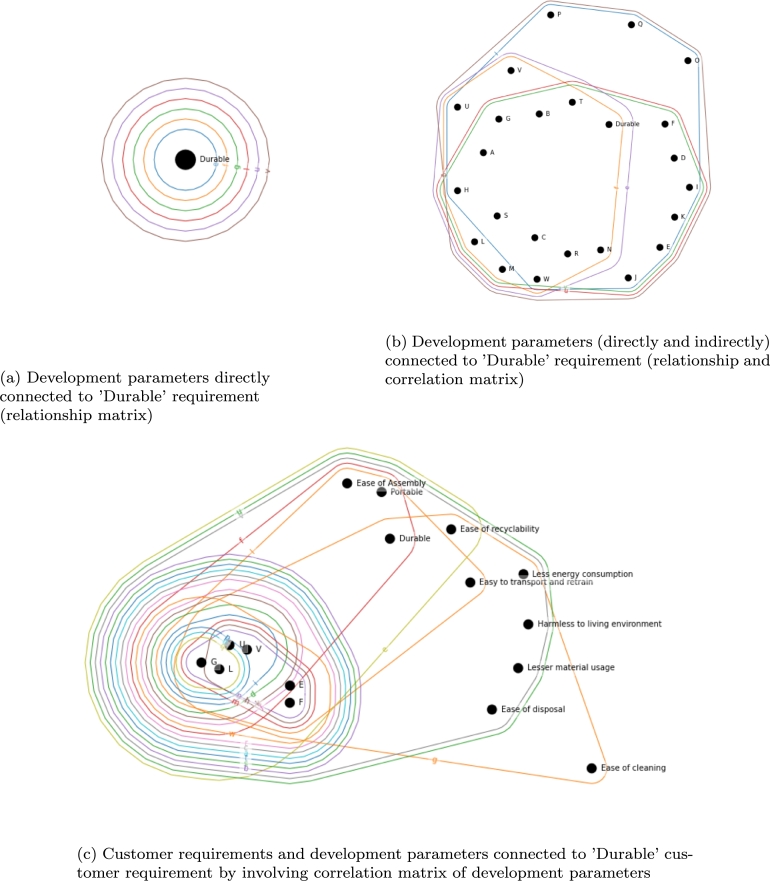
Hypergraph-based representation of the direct and indirect interactions in the House of Quality.

A representative interpretation of the joint implementation of relationship and correlation matrix is shown in Fig. 13. Fig. 13 indicates the connection between the ‘Durable’ customer requirement and the development parameters. In Fig. 13a the relationship matrix represents that there are six development parameters directly connected to this requirement, namely *e* - ‘Maintainability/Serviceability’, *f* - ‘Upgradability’, *g* - ‘Potential financial benefits’, *l* - ‘Installation and training cost’, *u* - ‘Weight of product’, *v* - ‘Volume of product’. The interaction between development parameters represented as shown in Fig. 13b. Vertices with capital letters are analog to the development parameters indicated with lower letters. This subfigure demonstrates that the six edges mentioned above (development parameter) correlate with which development parameters. For example, *f* - ‘Upgradability’ beyond the customer requirement ‘Durable’, it connects also development parameters a,b,c,g,h,l,m,n,r,s,t,u,v,w, from which a,b,c,h,m,n,r,s,t,u,w are only indirectly, through the correlation with this development parameter effect the satisfaction of this customer requirement. It nicely represents how interconnectedness of the development actions. Furthermore, the development of a parameter affects other customer requirements, and the indirectly involved development parameters can bring aspects that wouldn't be considered while analyzing only the relationship matrix. Fig. 13c represents that even in the case of requirement, the cross-relationship between the development parameters has an indirect effect on the other customer requirements. For example, by developing *f* - ‘Upgradability’, beyond ‘Durable’ customer requirement, the ‘Portable’ and ‘Ease of Assembly’ customer requirements are also affected, and due to the interconnection between the development parameters, those that interact with *f* also involves different customer requirements. There is a strong correlation between *f* and *a*, and due to that, *a* involves additional customer requirements: ‘Less energy consumption’, ‘Ease of recyclability’, ‘Lesser material use’, ‘Ease of disposal’, ‘Ease of cleaning’ and ‘Harmless to the living environment’. Therefore, the investment in *f* could also be beneficial to *a* and its connected customer requirements.

This method is useful in exploring relationships and visualizing the interactions between the elements of QFD. The centrality metrics allow for identifying critical/important elements worth focusing on.

### 4.3 Evaluation of the results

In this section, the comparison of the methods and their results is discussed. The performance of each model is compared according to their:

• Selected development parameters
• Total efficiency: It is the sum of $y_{i}$ values for all selected development parameter.
• Total cost: It is the weighted sum of selected development parameters with the cost related to the development parameter.
• Possible utilization areas

By implementing the correlation matrix directly into the calculation of importance ranking, changes in the QFD can be automatically represented and save time for the engineering team. The proposed methods contribute to creating the QFD, as they support evaluating the relationships and selecting the key development parameters in different strategic planning. Each method has different objectives, supporting various scenarios or development strategies. The application of linear programming-based QFD aims to maximize the effectiveness by the connection between the relationship and correlation matrix. It considers capacity constraints and can be utilized when we want to maximize the fulfillment of customer requirements under a finite, e.g., budget constraint. The assignment as a minimum cost flow approach seeks to minimize the costs of actions. This approach can be useful when investing in development parameters based on a cost-effective approach. The hypergraph-based representation of QFD supports great visualization and identification of the cross-relationships. The overlaps of the hyperedges indicate which development parameter is connected to the same customer requirement (and reverse). The generalized hypergraph allows for jointly analyzing the correlation and relationship matrices. It is an excellent tool for visualizing the direct and indirect interactions of customer requirements and development parameters.

Table 11 shows the comparison of the results based on the three approaches. Each method has selected the development parameter ‘d’ (Ease of use), while linear programming and minimum cost flow additionally selected ‘e’ (Maintainability/Serviceability), ‘f’ (Upgradability) and ‘k’ (Human toxicity), ‘m’ (Material utilization), ‘n’ (Energy efficiency/Power consumption) development parameters. The ‘u’ (Weight of product) was jointly selected by minimum cost flow and hypergraph.

**Table 11:** Comparison of the results based on the proposed methods.

	Linear programming	Minimum cost flow	Hypergraph
Objective	Maximize efficiency	Minimize cost	Visualization, centrality
Total efficiency value	33.679	30.809	29.619
Total cost of actions	27	18	36
Selected development parameters	**d,e,f**,i,j,**k**,l,**m,n**,r	**d,e,f,h,k,m,n**,o,p,**u**	a,b,c,**d**,g,**h**,t,**u**,v,w
Applicability/Development strategy	Greatest customer satisfaction	Cost-effective approach	Support all types

## 5 Conclusion

In this study, the potential improvement of the QFD method has been proposed to overcome the limitations of QFD and support engineering knowledge and reliable decision-making. The overview of QFD-related literature highlighted that, however, the general QFD considers correlation matrix of development parameters it serves more as additional information for the development team, and most of the extensions of QFD do not implement correlation matrix in their calculation. Research integrating the correlation matrix into their calculations are often specialized to a field. Furthermore, the group interactions between the elements of QFD have not been analyzed and visualized extensively. Therefore, the interactions between the development parameters and their indirect effect on customer requirements remain hidden or specialized, and optimal resource allocation cannot be made and generally applied for QFD.

This study promotes approaches that overcome the above mentioned drawbacks, and integrate the correlation matrix into their calculation as well as allow better analysis and visual representation of identifying cross-relationships. The characteristics of the proposed improvement methods of QFD are shown in Table 12. QFD is accessed as a network flow optimization problem with two objectives. One is to maximize the benefit of the selected development parameters under possible capacity constraints. The other is to minimize the total cost of actions but still meet the customer requirements and bring enough benefit. Then, QFD is accessed from the analysis of QFD as a hypergraph, which identifies central important elements of the graph and serves as an excellent tool for visualizing direct and indirect interactions between the elements.

**Table 12:** Characteristics of the proposed integrated methods for improving QFD.

QFD accessed from an:	Reverse approach of identifying importance of development parameters		

Advantages	- allows better identification of cross-relationships		
	- the calculation directly involves the correlation matrix of development parameters		
	- allows better identification of focus areas and investment potentials		

			

QFD considered as:	Maximizing the efficiency of actions	Assignment as minimum cost flow	Hypergraph representation

Advantage	- optimization method	- optimization method	- illustrative interpretation
	- maximize the fulfillment of customer requirements	- minimize costs	- utilization of network tools (e.g. centrality)
	- consider capacity constraint	- consider capacity constraint	- generalized hypergraph allows the implementation of correlation matrix

Development Potential	- consider the effect of time and uncertainty - implementation for cascade of houses		

Utilization Potential	- product planning and development strategy making, resource allocation plans		

We believe that the proposed aspects support product development by identifying the cross-relationships between the elements of the House of Quality and visualizing their interaction. As highlighted before, each method has a potential utilization based on the strategic aspect of the project. Thereby, it supports efficient resource allocation and strategy making in various fields due to the general formulation of the problem.

A possible future research direction is to consider uncertainty and the effect of time during the analysis. Furthermore, applying the proposed methods to the cascade of houses is a potential development direction so that the whole development project could be supported from the product planning phase until production planning and the cross-relationships between the phases could be unfolded.

## Declarations

### Author contribution statement

János Abonyi and Tímea Czvetkó: Conceived and designed the experiments; Performed the experiments; Analyzed and interpreted the data; Contributed reagents, materials, analysis tools or data; Wrote the paper.

### Funding statement

This work was supported by 10.13039/501100012550Nemzeti Kutatási, Fejlesztési és Innovaciós Alap [TKP2021-NVA-10], 10.13039/501100015498Innovációs és Technológiai Minisztérium [OTKA 143482 (Monitoring Complex Systems by goal-oriented clustering algorithms)].

### Data availability statement

Data included in article/supp. material/referenced in article.

### Declaration of interests statement

The authors declare no conflict of interest.

### Additional information

No additional information is available for this paper.
